# The psychosocial antecedents of the adherence to the Mediterranean diet

**DOI:** 10.1017/S1368980022000878

**Published:** 2022-04-13

**Authors:** Valentina Carfora, Maria Morandi, Anđela Jelić, Patrizia Catellani

**Affiliations:** Department of Psychology, Catholic University of the Sacred Heart, Largo Agostino Gemelli, 1, Milan 20123, Italy

**Keywords:** Mediterranean diet, Attitude, Perceived behavioural control, Subjective norm, Health motive, Mood motive, Anticipated emotions

## Abstract

**Objective:**

Most previous research on the antecedents of healthy food choice has not investigated the links between these antecedents and has focused on specific food choice rather than on an overall diet. In the present study, we tested the plausibility of an integrated theoretical model aiming to explain the role of different psychosocial factors in increasing the intention to adhere to the Mediterranean Diet (MeDiet).

**Design:**

An online survey measured participants’ attitude and perceived behavioural control (i.e. rational antecedents), subjective norm (i.e. social antecedent), positive and negative anticipated emotions (i.e. emotional antecedents), food choice health and mood motives (i.e. motivational antecedents), past adherence to the MeDiet (i.e. behavioural antecedent), and intention to adhere to the MeDiet.

**Setting:**

Italy.

**Participants:**

1940 adults: 1086 females; 854 males; mean age = 35·65; sd = 14·75; age range = 18–84.

**Results:**

Structural Equation Modelling (sem) analyses confirmed the plausibility of the proposed model. Perceived behavioural control was the strongest rational antecedent of intention, followed by the emotional (i.e. anticipated emotions) and the social (i.e. subjective norm) antecedents. Mediation analysis showed that motivational antecedents had only an indirect impact on intention via emotional antecedents. Finally, multigroup sem analysis highlighted that past adherence to the MeDiet moderated the hypothesised paths among all the study variables.

**Conclusions:**

The above findings advance our comprehension of which antecedents public communication might leverage to promote an increase in the adherence to the MeDiet.

The Mediterranean Diet (MeDiet) is a traditional dietary pattern distinctive of the Mediterranean olive cultivation areas^(1)^. It received the recognition of UNESCO Intangible Cultural Heritage in 2010, as it contributes to transmit a set of knowledge, symbols and rituals regarding food production, conservation, cooking and consumption, which is the basis of the cultural identity and continuity of the Mediterranean communities^(2)^. The MeDiet is characterised by the consumption of a variety of fresh, local and seasonal food products. Its recommended dietary pattern is represented by the MeDiet Pyramid, which is divided into foods that should be consumed on a daily, weekly and monthly basis. Specifically, plant foods (i.e. cereals, fruit, vegetables, legumes and nuts) and olive oil should be eaten daily. Dairy products, fish, seafood, eggs, cheese, yogurt and poultry should be consumed weekly. Finally, sweets, red meat and processed meat should be preferably consumed monthly^(1)^.

An increasing number of studies supports the beneficial effects of the MeDiet on a range of physical and mental health outcomes. For instance, the MeDiet shows a general preventive effect against CVD, obesity, metabolic syndrome, diabetes, several types of cancer, osteoporosis and premature mortality^(3,4)^. The MeDiet is also beneficial for brain functioning, as it is a protection factor against cognitive decline and dementia, Parkinson’s disease and depression^(3)^. Furthermore, as the MeDiet is related to positive health outcomes and quality of life^(5)^, an increase of the adherence to the MeDiet might reduce private and societal health-related costs^(6)^. For all these reasons, many national and European organisations and institutions are currently trying to support and pursue the values and benefits of the MeDiet.

Despite this public effort, an increase of the westernisation of nutritional habits is leading to a lower adherence to the MeDiet in both Mediterranean and Non-Mediterranean countries^(7)^. In Italy, for example, nutritional habits are scarcely consistent with the MeDiet recommendations, with more than half of Italians having a low consumption of plant foods and a high consumption of sweets, red and processed meat^(8)^.

So far, research on the psychosocial antecedents that might lead people to have a higher adherence to the MeDiet recommendations have been scarce. To overcome this gap in the current literature, in the present study, we aimed to propose and test the plausibility of an integrated theoretical model to assess the role of different psychosocial (rational, social, emotional, motivational and behavioural) antecedents in increasing the intention to adhere to the MeDiet. The identification of these psychosocial antecedents and their relationships is important to design effective public and private promotion campaigns aimed at increasing the adoption of the MeDiet.

## Theoretical background

**Fig. 1:**
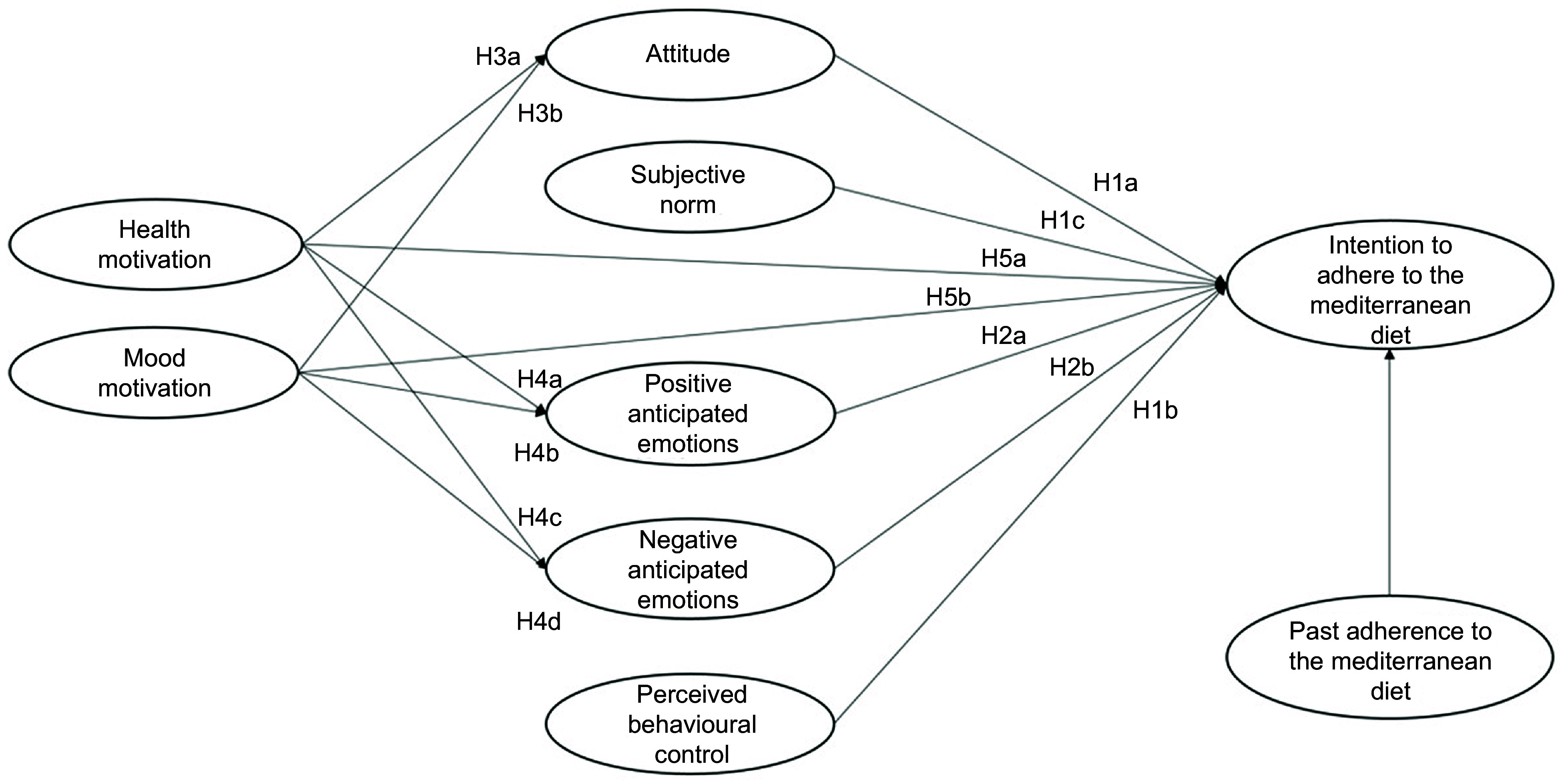
Integrated theoretical model to explain the intention to adhere to the Mediterranean diet

Previous research on the antecedents of healthy food choice has mainly focused attention mainly on the rational components of this choice, such as attitude, perceived behavioural control or self-efficacy^(9,10)^. A more limited number of studies have instead investigated the motivational, emotional and behavioural components of this choice, such as health motives or habits^(11,12)^. So far, however, the links between these antecedents have been scarcely investigated, and this is likely due to the lack of a common conceptual framework to understand the predictors of food choice and diet^(13)^. Moreover, most of the past studies focused on the reasons for choosing individual foods (e.g. fruit and vegetable, meat, sugary snack or beverage) rather than considering the difficult issue of predicting adherence to a healthy diet, which indeed is the key to obtaining long-term health benefits^(14)^. In the present study, we proposed and tested an integrated model of the different antecedents that influence the adherence to the MeDiet, by considering rational, social, emotional, motivational and behavioural antecedents. These antecedents are presented in Fig. 1 and described below, together with the hypotheses about their interrelationships.

To consider the *rational and social antecedents* of people’s intention to adhere to the MeDiet, we assumed the Theory of Planned Behaviour (TPB) as a frame of reference^(15)^. The TPB postulates that the personal intention to perform a behaviour is determined by both rational and social factors. The rational factors include attitude and perceived behavioural control, where *attitude* is related to the personal positive or negative evaluation of a behaviour, while *perceived behavioural control* is the perception of personal ability and external possibility to perform a specific behaviour. The social component of the TPB model is represented by *subjective norm*, that is the perceived social pressure to perform a certain behaviour.

In the domain of the studies on eating behaviours, several meta-analyses confirmed that the TPB is a robust theoretical model to predict people’s intentions related to food choice^(16)^, as well as the intention to follow a healthy dietary pattern^(9)^. To the best of our knowledge, however, only few studies have explored and confirmed the predictive power of the TPB on the adherence to the MeDiet^(13,17)^. In these studies, perceived behavioural control appeared to be the strongest predictor of the intention to adhere to the MeDiet, followed by a positive attitude towards the MeDiet. Subjective norm had instead the lowest role in predicting people’s intention. Based on the above, in the present study, we expected that:

**H1a**: Positive attitude towards the adherence to the MeDiet influences the intention to adhere to it.

**H1b**: Perceived behavioural control influences the intention to adhere to the MeDiet.

**H1c**: Subjective norm related to the adherence to the MeDiet influences the intention to adhere to it.

Over and above the rational and social factors, *emotional antecedents* are likely to play a relevant role in orienting diet habits. Emotions are essential elements in the decision-making process, as they influence information processing, responses to persuasive stimuli, goal setting and goal-directed behaviour implementation^(18)^. Among the different types of emotions, *anticipated emotions* have aroused considerable interest given their strong predictive power. They can be defined as the positive or negative emotional reactions elicited by the anticipation of the consequences of acting or not acting a certain behaviour^(19)^.

Many researchers have already included anticipated emotions in the TPB model, even if most of them have paid attention only to anticipated emotions with a negative valence (e.g. anticipated regret, guilt or shame), without considering those with a positive valence (e.g. satisfaction, happiness or proudness)^(20)^. Moreover, so far, only few studies have included anticipated emotions in theoretical models aimed to explain food choice^(21–23)^, and again they focused predominantly on negative anticipated emotions. To the best of our knowledge, only Mari *et al.*^(13)^ have investigated the role of anticipated emotions in the prediction of the adherence to the MeDiet. This study has shown that both positive and negative anticipated emotions influence the desire to follow the MeDiet. Thus, in our study, we hypothesised that:

**H2a**: Positive anticipated emotions related to following the MeDiet influence the intention to adhere to the MeDiet.

**H2b**: Negative anticipated emotions related to following the MeDiet influence the intention to adhere to the MeDiet.

*Motivational antecedents* also play a relevant role in determining people’s food choices, and these antecedents include health and mood motives^(11)^. On the one side, *health motive* leads individuals to choose healthy and nutritious food (e.g. rich in vitamins and minerals) or food that improves physical appearance (e.g. promote skin, teeth, hair, nails appearance). On the other side*, mood motive* is centred on the emotional well-being and highlights individuals’ interest to eat food to reduce stress and relax, or to cheer up and feel good^(11)^. These motives play a decisive role in influencing food choice insofar as they determine people’s attitude towards the selection of certain foods. For instance, they have been found to influence attitude towards healthy eating and personalised dietary advice^(24,25)^. Although the role of these motives in the case of people’s intention to adhere to the MedDiet has not been analysed, starting from the results of past studies on motivational antecedents of other healthy diets in the present study we expected that:

**H3a**: *Health motive* influences positive attitude towards the MeDiet.

**H3b**: *Mood motive* influences positive attitude towards the MeDiet.

When evaluating a behaviour, people feel positive or negative emotions as a consequence of the perception of consistency or inconsistency of this behaviour with their inner motivations. This emotional experience improves the ability to anticipate similar emotions when deciding how to act in the future and, in turn, influences intentions^(26)^. Similarly, when making a food choice, people are likely to mentally anticipate the emotions they will experience if such choice is either consistent or not consistent with their health or mood motives. Accordingly, in the present study, we tested the hypotheses below.

**H4a**: Health motive elicit positive anticipated emotions towards adhering to the MeDiet.

**H4b**: Health motive elicit negative anticipated emotions towards adhering to the MeDiet.

**H4c**: Mood motive elicit positive anticipated emotions towards not adhering to the MeDiet.

**H4d**: Mood motive elicit negative anticipated emotions towards not adhering to the MeDiet.

Motivational antecedents have been shown to have also a direct impact on intention related to food choice. For example, health motive has been found to directly predict the intention to purchase both healthy and unhealthy food^(27,28)^. Similarly, mood motive has been found to directly influence the intention to follow personalised dietary advice^(24)^. To the best of our knowledge, no previous research has investigated the role of health and mood motives to predict the adherence to the MeDiet. In the present study, we tested the following hypotheses.

**H5a**: Health motive influences the intention to adhere to the MeDiet.

**H5d**: Mood motive influences the intention to adhere to the MeDiet.

Finally, in our study, we considered the *behavioural antecedents* of the intention to adhere to the MeDiet. Several studies have shown that *past behaviour* is a powerful predictor of behaviour intention^(12,29,30)^. Actually, several researchers have included past behaviour in the TPB model, showing that it is often the strongest predictor of intention, over and above the original TPB variables. This evidence has been confirmed also in the case of frequently repeated behaviours^(31)^. Previous scholars have shown that the inclusion of past behaviour increases the predictiveness of the TPB model to explain people’s intention related to frequent food choices^(32,33)^, also in the case of people’s intention to follow the MeDiet^(13)^. Accordingly, we hypothesised that:

**H6**: Past adherence to the MeDiet influences the intention to follow the MeDiet.

Previous researchers have also found that past behaviour is a moderator within the framework of the TPB. For example, past behaviour was shown to moderate the attitude-intention and the anticipated emotion-intention relationships^(31,34)^. Consistently, in the present study, we aimed to evaluate whether different levels of past adherence to the MeDiet (low, medium and high adherence) would produce a different impact of the other study variables on participants’ intention to adhere to the MeDiet. Hence, we investigated the Research Question (RQ) below.

**RQ**: Past behaviour moderates the impacts of the other study variables on the intention to adhere to the MeDiet.

## Method

### Participants and measures

Ethical approval for this study was obtained from the Catholic University of the Sacred Heart (Milan). Using the A-priori Sample Size Calculator for Structural Equation Models created by Daniel Soper^(35)^, we computed the minimum sample size required for a structural equation model study. Results recommended to involve at least 184 participants to test our integrated model (nine latent variables and thirty observed variables; expected effect size = 0·30; *P*-value = 0·05; statistical power level = 0·80) and 243 participants to test the moderation of the three levels of past adherence to the MeDiet (twenty-seven latent variables and ninety observed variables; expected effect size = 0·30; *P* value = 0·05; statistical power level = 0·80). However, we opted to increase our sample size, following Jackson’s recommendation^(36)^ to have a sample size to parameters ratio of 20:1 or at least 10:1.

In October 2020, about 2100 Italian adults were invited by the students of the Department of Psychology to fill in an online questionnaire. Each student was asked to invite three females (one between the age of 18 and 25, one between the age of 26–45, one between the age of 46–75) and three males (same criteria used for females) via e-mail or text message. At the beginning of the questionnaire, we provided participants with instructions on how to properly fill out the online questionnaire, as well as information on how to employ the different types of measurement scales. A control question to verify if participants’ replies were reliable was also included in the questionnaire. A total of 1940 participants correctly and fully completed the questionnaire (1086 females; 854 males; mean age = 35·65; sd = 14·75; age range = 18–84). A description of the measures collected through the questionnaire follows below. The full list of the items of each measure is reported in Table 1.

**Table 1:** Results of the measurement model

Construct	Items	*λ*	CR	AVE
Health motive	It is important that the food I eat…		0·88	0·71
	… contains vitamins and minerals	0·77		
	… is healthy	0·86		
	… is nutritious	0·63		
Mood motive	It is important that the food I eat…		0·91	0·77
	… helps me relax	0·78		
	… cheers me up and makes me feel good	0·77		
	… helps me cope with stress	0·89		
Attitude towards the MeDiet	In the next month, following the MeDiet will be/would be…		0·95	0·70
	… disgusting – tasty	0·74		
	… sad – joyful	0·69		
	… unpleasant – pleasant	0·74		
	… boring – funny	0·61		
	… disadvantageous – advantageous	0·88		
	… fool – wise	0·91		
	… uneffective – effective	0·91		
	… useless – useful	0·91		
Perceived behavioural control	In the next month…		0·86	0·67
	… following the MeDiet will be entirely up to me	0·50		
	… I think I have enough opportunities to follow MeDiet	0·82		
	… I feel capable of following the MeDiet	0·80		
Subjective norm	Most of the people who are important to me…		0·89	0·73
	… think that I should follow the MeDiet	0·93		
	… would approve if I followed the MeDiet in the next month	0·94		
	… would like me to follow the MeDiet in the next month	0·47		
Positive anticipated emotions	In the next month, if I will follow MeDiet …		0·93	0·82
	… I will be proud of myself	0·85		
	… I will feel secure	0·82		
	… I will be satisfied	0·89		
Negative anticipated emotions	In the next month, if I will not follow the MeDiet …		0·94	0·84
	… I will regret it	0·84		
	… I will feel worried	0·88		
	… I will feel dissatisfied	0·88		
Intention to adhere to the MeDiet	In the next month…		0·97	0·92
	… I intend to follow the MeDiet	0·92		
	… I plan to follow the MeDiet	0·94		
	… I will follow the MeDiet	0·95		

*λ*, standardised factor loading; CR, composite reliability; AVE, average variance extracted; MeDiet, Mediterranean diet.

Attitude was assessed with a seven-point semantic differential scale. All other scales were measured with a response scale varying from ‘strongly disagree’ (1) to ‘strongly agree’ (7).

#### Health motive

Participants’ health motive regarding food choice was measured with three items on a seven-point Likert scale (e.g. ‘It’s important that the food I eat contains vitamins and minerals… strongly disagree (1) – strongly agree (7)’; adapted from Naughton *et al*.^(11)^). High scores indicated a strong health motive regarding food choice (*α* = 0·79).

#### Mood motive

Participants’ mood motive regarding food choice was measured with three items on a seven-point Likert scale (e.g. ‘It’s important that the food I eat helps me relax… strongly disagree (1) – strongly agree (7)’; adapted from Naughton *et al*.^(11)^). High scores showed a high mood motive regarding food choice (*α* = 0·76).

#### Attitude

Using a seven-point semantic differential scale with eight items, we measured participants’ attitudes towards the MeDiet (e.g. ‘Following the Mediterranean Diet is…unpleasant – pleasant’; adapted from Mari *et al.*^(13)^). High values indicated a greater positive attitude towards the MeDiet (*α* = 0·94).

#### Subjective norm

Participants’ subjective norm was measured using three items on a seven-point Likert scale (e.g. ‘Most people who are important to me think that I should follow the Mediterranean Diet… strongly disagree (1) – strongly agree (7)’; adapted from Mari *et al*.^(13)^). High scores showed a elevate perception of a social expectation towards following the MeDiet (*α* = 0·81).

#### Perceived behavioural control

Participants’ perceived behavioural control over following the MeDiet was assessed using three items on a seven-point Likert scale (‘In the next month, following the Mediterranean Diet will be entirely up to me … strongly disagree (1) – strongly agree (7)’; adapted from Mari *et al*.^(13)^). High scores indicated a high perception of control over adhering to the MeDiet (*α* = 0·94).

#### Anticipated positive emotions

Participants’ anticipated positive emotions for following the MeDiet were measured using three items (e.g. ‘In the next month, if I will follow the Mediterranean Diet I will be proud of myself… strongly disagree (1) – strongly agree (7)’; adapted form Carfora *et al*.^(37)^). High score indicated strong anticipated positive emotions (*α* = 0·94).

#### Anticipated negative emotions

Participants’ anticipated negative emotions for not following the MeDiet were measured using three items (e.g. ‘In the next month, if I will not follow Mediterranean Diet I will regret it… strongly disagree (1) – strongly agree (7)’; adapted form Carfora *et al*.^(37)^). High values indicated strong anticipated negative emotions (*α* = 0·81).

#### Past adherence to the MeDiet

Participants’ past adherence to the MeDiet was assessed using the ‘Short Questionnaire to Assess Adherence to the Mediterranean Diet’^(38)^ composed of fourteen items (e.g. ‘How many fruit portions (including natural fruit juices) do you consume per day?… less than a portion (1) – more than five portions (7)’). The final score ranged from 1 to 14 and was obtained by recoding the responses in 0 points or 1 point following the criteria of the original scale.

#### Intention to adhere to the MeDiet

Participants’ intention to adhere to the MeDiet was assessed with three items on a seven-point Likert Scale (e.g. ‘In the next month, I intend to follow the Mediterranean diet … strongly disagree (1) – strongly agree (7)’; adapted from Mari *et al*.^(13)^). High scores showed a high intention to adhere to the MeDiet (*α* = 0·96).

At the beginning of the online questionnaire, participants provided written consent. Then, they completed two scales assessing health and mood motives related to their food choice and self-reported their adherence to the MeDiet. After that, they read a definition of the MeDiet (see Appendix) and filled out the TPB scales (attitude, subjective norm, perceived behavioural control) plus the scales to assess their positive and negative anticipated emotions regarding adherence to MeDiet. Finally, participants answered socio-demographic questions.

### Data analyses

We ran all analyses using MPLUS 7. As preliminary analyses, we tested the measurement model with confirmatory factor analysis. We verified the internal consistency among the observed variables using Cronbach’s *α* and composite reliability. We then tested convergent and discriminant validities of our data using average variance extracted (AVE) values.

Then, we verified our hypotheses (H1–H6) by testing the goodness-of-fit of four nested sem models. We compared the nested models with the *χ*^2^ difference test (Δ*χ*^2^). Each nested model is described below.

Model 1 tested our H1a–H1c about the role of rational and social antecedents and included the paths from attitude, perceived behavioural control and subjective norm to intention as free parameters. In this Model 1, the regression weights of the paths among the other variables were fixed to 0.

Model 2 tested our H2a and H2b on the emotional antecedents, by including the paths from positive and negative anticipated emotions to intention. The regression weights of the other hypothesised paths were fixed to 0.

Model 3 tested our H3–H6 related to the inclusion of motivational antecedents. Thus, we inserted the following paths: (a) the path from both health motive (H3a) and mood motive (H3b) to attitude; (b) the paths from both health motive (H4a) and mood motive (H4b) to positive anticipated emotions; (c) the paths from both health motive (H4c) and mood motive (H4d) to negative anticipated emotions intention; and (d) the paths from both health motive (H5a) and mood motive (H5b) to negative anticipated emotions intention.

The regression weights of the paths related to past adherence to the MeDiet were fixed to 0.

Model 4 verified our H6 about the inclusion of a behavioural antecedent by including path from past adherence to the MeDiet to intention.

Finally, to test our RQ, we run a multigroup sem analysis to verify if our integrated model (Model 4) would differ according to the past adherence to the MeDiet. We created a group variable to distinguish among the *low-adherence* group, the *medium-adherence group* and the *high-adherence* group^(38)^. The low-adherence group included participants with a score equal to or < 5 on the past adherence variable. The medium-adherence group included participants with a score from 6 to 9 on the past adherence variable. The high-adherence group included participants with a score equal to or greater than 10 on the past adherence variable. Then, to disconfirm the invariance of the paths among the study variables across the above groups, we constrained the paths of each group to be invariant in the other groups, and then we used Wald tests to disconfirm the invariance of the paths. These analyses allowed us to verify if the past adherence to the MeDiet moderated the relationship among the psychosocial antecedents and the participants’ intention to follow the MeDiet in the following month.

In all the above analyses, the goodness of fit of all models was tested using *χ*^2^ and incremental goodness-of-fit indexes: root mean square error of approximation (RMSEA) < 0·05, comparative fit index (CFI) < 0·90, Tucker-Lewis index (TLI) < 0·90 and standardised root mean squared residual < 0·08^(39,40)^. Models with significant *χ*^2^ test results were accepted on the condition that the CFI or TLI value reaches 0·95 or more, and the value of RMSEA was fewer than 0·08^(41)^.

## Results

### Preliminary analyses

All socio-demographic data are reported in Table 2. Confirmatory factor analysis showed that the measurement model fit the data satisfactorily (*χ*^*2*^ (349) = 4320·16, *P* = 0·001; RMSEA = 0·07, CFI = 0·91, TLI = 0·90, standardised root mean squared residual = 0·05). Composite reliability values were all greater than the minimum threshold of 0·60^(42)^. Thus, we confirmed the reliability of the measurement model. The standardised factor loadings and the AVE values were all above the recommended threshold^(43,44)^, showing that all constructs had a high convergent validity. Finally, all AVE were higher than correlations between latent constructs, confirming the discriminant validity of the study variables^(44)^. Table 1 shows the results of the measurement model. Table 3 reports means, SD and AVE of our study variables and correlations among them.

**Table 2:** Demographics of study sample

	Female	Male	Total
Age			
% 18–24 years (young)	34·7	29·4	32·4
% 25–34 years (young adults)	22·6	24·9	23·6
% 35–54 years (adults)	29·9	30·0	29·9
& 55+ (seniors)	12·8	15·7	14·1
Number of residents in your municipality			
% Less than 5·000	18·5	17·9	18·2
% Between 5000 and 30 000	42·6	41·8	42·3
% Between 30 000 and 250 000	12·0	10·1	11·1
% Between 250 000 and 500 000	21·0	23·9	22·3
% More than 500 000	5·9	6·3	6·1
Marital status			
% Single	18·5	17·9	50·6
% Married	42·6	41·8	31·8
% Cohabiting couple	12·0	10·1	11·9
% Separated/divorced	21·0	23·9	4·3
% Widow	5·9	6·3	1·5

**Table 3:** Means, standard deviations, average variance extracted values and correlations among the study variables

Study variables	1.	2.	3.	4.	5.	6.	7.	8.	9.	Mean	sd
1. Health motive	0·71									5·45	1·06
2. Mood motive	0·34	0·77								5·26	1·14
3. Attitude towards the MeDiet	0·21*	0·10*	0·70							5·55	1·32
4. Perceived behavioural control	0·29*	0·15*	0·32*	0·67						5·39	1·11
5. Subjective norm	0·13*	0·11*	0·20*	0·24*	0·73					1·40	3·98
6. Positive anticipated emotions	0·26*	0·24*	0·43*	0·31*	0·44*	0·82				5·50	1·11
7. Negative anticipated emotions	0·21*	0·17*	0·27*	0·46*	0·30*	0·42*	0·92			1·52	4·22
8. Past adherence to the MeDiet	0·27*	0·08*	0·19*	0·05*	0·25*	0·13*	0·11*	–		6·82	2·08
9. Intention to adhere to the MeDiet	0·31*	0·17*	0·40*	0·44*	0·56*	0·57*	0·50*	0·25*	0·92	4·73	1·50

sd = standard deviation.

* *P* = 0·001.

In the diagonal row, the bold values are the average variances extracted for the latent construct.

The numbers below diagonal are the correlation coefﬁcients among the study variables.

### Model comparisons

The results of the comparisons among the four nested models showed that only model 4 (i.e. the model including attitude, perceived behavioural control, subjective norm, positive and negative anticipated emotions, health and mood motives and past adherence to the MeDiet as predictors of participants’ intention to adhere to the MeDiet) had an acceptable goodness of fit (Model 4: *χ*^*2*^ (369) = 2837·460, *P* = 0·001; RMSEA = 0·05, CFI = 0·92, TLI = 0·91, standardised root mean squared residualc = 0·05). The comparison between model 1 and model 2 supported the addition of positive and negative anticipated emotions, Δ*χ*^2^ (9) = 1364·21, *P* = 0·001. The comparison between model 2 and model 3 confirmed the inclusion of health and mood motives, Δ*χ*^2^ (13) = 407·70, *P* = 0·001. Finally, the comparison between model 3 and model 4 supported the inclusion of past adherence to the MeDiet, Δ*χ*^2^ (8) = 234·75, *P* = 0·001. Therefore, as expected, the more comprehensive model 4 was the model that best predicted participants’ intentions to adhere to the MeDiet. Table 4 shows the goodness of fit and the standardised coefficients of each tested model.

**Table 4:** Goodness of fit and standardised coefficients for each nested model

	Model 1 (TPB)	Model 2 (model 1 plus anticipated emotions)	Model 3 (model 2 plus motivations)	Model 4 (model 3 plus past adherence)
*χ*^2^ (df)	4844·12 (399); *P* = 0·001	3479·91 (390); *P* = 0·001	3072·21 (377); *P* = 0·001	2837·46 (369); *P* = 0·001
RMSEA	0·09	0·06	0·06	0·05
CFI	0·87	0·90	0·91	0·92
TLI	0·86	0·89	0·90	0·91
SRMR	0·19	0·11	0·06	0·05
Attitude towards the MeDiet → intention to adhere to the MeDiet	0·12**	0·04*	0·03	0·03
Perceived behavioural control → intention to adhere to the MeDiet	0·55**	0·43**	0·42**	0·41**
Subjective norm → intention to adhere to the MeDiet	0·28**	0·18**	0·18**	0·18**
Positive anticipated emotions → intention to adhere to the MeDiet	Fixed to 0	0·21**	0·21**	0·21**
Negative anticipated emotions → intention to adhere to the MeDiet	Fixed to 0	0·18**	0·18**	0·18**
Health motive → attitude towards the MeDiet	Fixed to 0	Fixed to 0	0·17**	0·17**
Health motive → positive anticipated emotions	Fixed to 0	Fixed to 0	0·23**	0·23**
Health motive → negative anticipated emotions	Fixed to 0	Fixed to 0	0·20**	0·20**
Health motive → intention to adhere to the MeDiet	Fixed to 0	Fixed to 0	0·06*	0·04
Health motive → attitude towards the MeDiet → intention to adhere to the MeDiet	Fixed to 0	Fixed to 0	0·01	0·00
Health motive → positive anticipated emotions → intention to adhere to the MeDiet	Fixed to 0	Fixed to 0	0·05**	0·05**
Health motive → negative anticipated emotions→ intention to adhere to the MeDiet	Fixed to 0	Fixed to 0	0·03**	0·03**
Mood motive → attitude towards the MeDiet	Fixed to 0	Fixed to 0	0·03	0·03
Mood motive → positive anticipated emotions	Fixed to 0	Fixed to 0	0·19**	0·19**
Mood motive → negative anticipated emotions	Fixed to 0	Fixed to 0	0·13**	0·13**
Mood motive → intention to adhere to MeDiet	Fixed to 0	Fixed to 0	0·02	0·02
Mood motive → attitude towards the MeDiet → intention to adhere to the MeDiet	Fixed to 0	Fixed to 0	0·00	0·00
Mood motive → positive anticipated emotions → intention to adhere to the MeDiet	Fixed to 0	Fixed to 0	0·04**	0·04**
Mood motive → negative anticipated emotions→ intention to adhere to theMeDiet	Fixed to 0	Fixed to 0	0·02**	0·02**
Past adherence to the MeDiet → intention to adhere to the MeDiet	Fixed to 0	Fixed to 0	Fixed to 0	0·06**
*R*^2^ attitude towards the MeDiet	–	–	0·03**	0·03**
*R*^2^ positive anticipated emotions	–	–	0·12**	0·12**
*R*^2^ negative anticipated emotions	–	–	0·07**	0·07**
*R*^*2*^ intention to adhere to the MeDiet	0·57**	0·62**	0·63**	0·64**

MeDiet, Mediterranean diet; *χ*^2^, goodness-of-fit statistics; df, degrees of freedom of *χ*^2^ statistics; CFI, comparative fit index; TLI , Tucker–Lewis fit index; RMSEA, root mean square error of approximation.

* *P*< 0·05.

** *P* < 0·001.

**Fig. 2:**
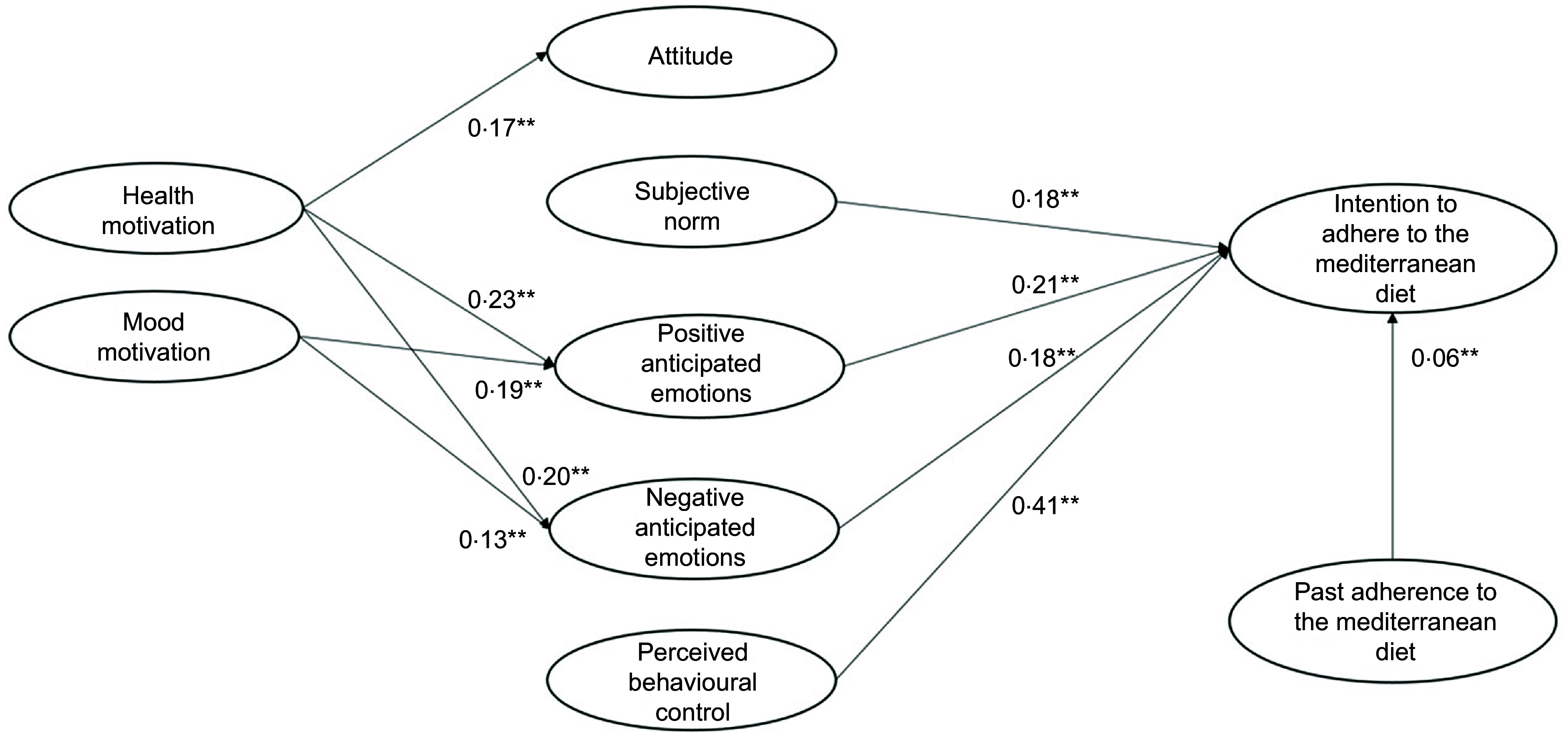
Results of the integrated model to explain the intention to adhere to the Mediterranean diet (model 4)

Regarding the predictiveness of the rational and social antecedents, Model 4 (Table 4 and Fig. 2) showed that participants’ attitude did not predict the intention to adhere to the MeDiet, disconfirming our H1a. It should be noted that before the addition of the health and mood motivations, attitude was a significant predictor of participants’ intention (model 1: *β* = 0·12, *P* = 0·001; model 3: *β* = 0·04, *P* = 0·05). Our H1b and H1c were instead confirmed, with both perceived behavioural control and subjective norm having a significant effect on participants’ intention.

As regards the contribution of the emotional antecedents in explaining the intention to adhere to the MeDiet, both positive and negative anticipated emotions explained participants’ intention. Therefore, we confirmed our H2a and H2b.

We then tested the role of the motivational antecedents and found that health motive influenced attitude, positive anticipated emotions and negative anticipated emotions. Thus, our H3a, H4a and H4c were confirmed. Instead, health motive did not explain participants’ intention, disconfirming our H5a. Additional mediation analyses showed that health motives had an indirect impact on intention via both positive and negative anticipated emotions, but not via attitude.

Regarding the effect of participants’ mood motive, this variable did not predict participants’ positive attitude, thus we rejected our H3b. However, mood motive determined both positive and negative anticipated emotion, confirming our H4b and H4d. Similar to health motive, mood motive did not explain participants’ intention, and thus H5b was not supported. As for health motive, mood motive had an indirect impact on intention via both positive and negative anticipated emotions, but not via attitude.

Finally, the inclusion of the path of past adherence to the MeDiet on participants’ intention was supported, confirming H6 and the importance of considering behavioural antecedents related to past adherence to the MeDiet.

In model 4, the variances of attitude (*R*^2^ = 0·03), positive anticipated emotions (*R*^2^ = 0·12), negative anticipated emotions (*R*^2^ = 0·07) and intention (*R*^2^ = 0·64) were all significantly explained.

In sum, the above results confirmed the importance of integrating rational, social, emotional, motivational and behavioural factors to explain intention to adhere to the MeDiet. Moreover, they suggested that participants’ perception of control over following the MeDiet was the rational antecedent that most influenced participants’ intention, followed by both emotional and social antecedents. Finally, the motivational antecedents only indirectly determined participants’ intention by increasing their anticipation of positive and negative emotions deriving from adherence or missed adherence to the MeDiet.

### Comparison of the integrated model across low, medium and high levels of adherence to the MeDiet

Multigroup sem analysis was used to investigate differences in the impact of the study variables on participants’ intention to adhere to the MeDiet across each group of MeDiet adherence (low, medium and high adherence). The paths among the study variables were the same of those tested in model 4. The multi-group models obtained an acceptable fit (*χ*^*2*^ = 4007·12, df = 1191; *χ*^*2*^ contribution of the Low-Adherence Model = 1332·49; *χ*^*2*^ contribution of the Medium-Adherence Model = 1892·23; *χ*^*2*^ contribution of the High-Adherence Model = 782·40; RMSEA = 0·06; CFI = 0·91; TLI = 0·90; Table 5).

**Table 5:** Standardised factor loadings in the case of low, medium and high adherence to the MeDiet

	Low-adherence group	Medium-adherence group	High-adherence group
Attitude towards the MeDiet → intention to adhere to the MeDiet	0·09*	0·02	0·00
Perceived behavioural control → intention to adhere to the MeDiet	0·48**	0·40**	0·30**
Subjective norm → intention to adhere to the MeDiet	0·23**	0·14**	0·28**
Positive anticipated emotions → intention to adhere to the MeDiet	0·02	0·28**	0·36**
Negative anticipated emotions → intention to adhere to the MeDiet	0·21**	0·18**	0·17**
Health motive → attitude towards the MeDiet	0·17**	0·10*	0·19*
Health motive → positive anticipated emotions	0·22**	0·22**	0·14
Health motive → negative anticipated emotions	0·18**	0·17**	0·12
Health motive → intention to adhere to the MeDiet	0·10*	0·02	0·02
Health motive → attitude towards the MeDiet → intention to adhere to the MeDiet	0·01	0·00	0·00
Health motive → positive anticipated emotions → intention to adhere to the MeDiet	0·00	0·06**	0·05
Health motive → negative anticipated emotions → intention to adhere to the MeDiet	0·04*	0·03**	0·02
Mood motive → attitude towards the MeDiet	0·01	0·05	0·04
Mood motive → positive anticipated Emotions	0·17**	0·20**	0·21*
Mood motive → negative anticipated emotions	0·09	0·14**	0·16*
Mood motive → intention to adhere to MeDiet	0·04	0·04	0·05
Mood motive → attitude towards the MeDiet → intention to adhere to the MeDiet	0·02	0·00	0·00
Mood motive → positive anticipated emotions → intention to adhere to the MeDiet	0·00	0·05**	0·07**
Mood motive → negative anticipated emotions → intention to adhere to the MeDiet	0·02	0·03**	0·03
Past adherence to the MeDiet → intention to adhere to the MeDiet	−0·04	0·05	0·05

MeDiet, Mediterranean diet.

* *P* < 0·05.

** *P* < 0·001.

**Fig. 3:**
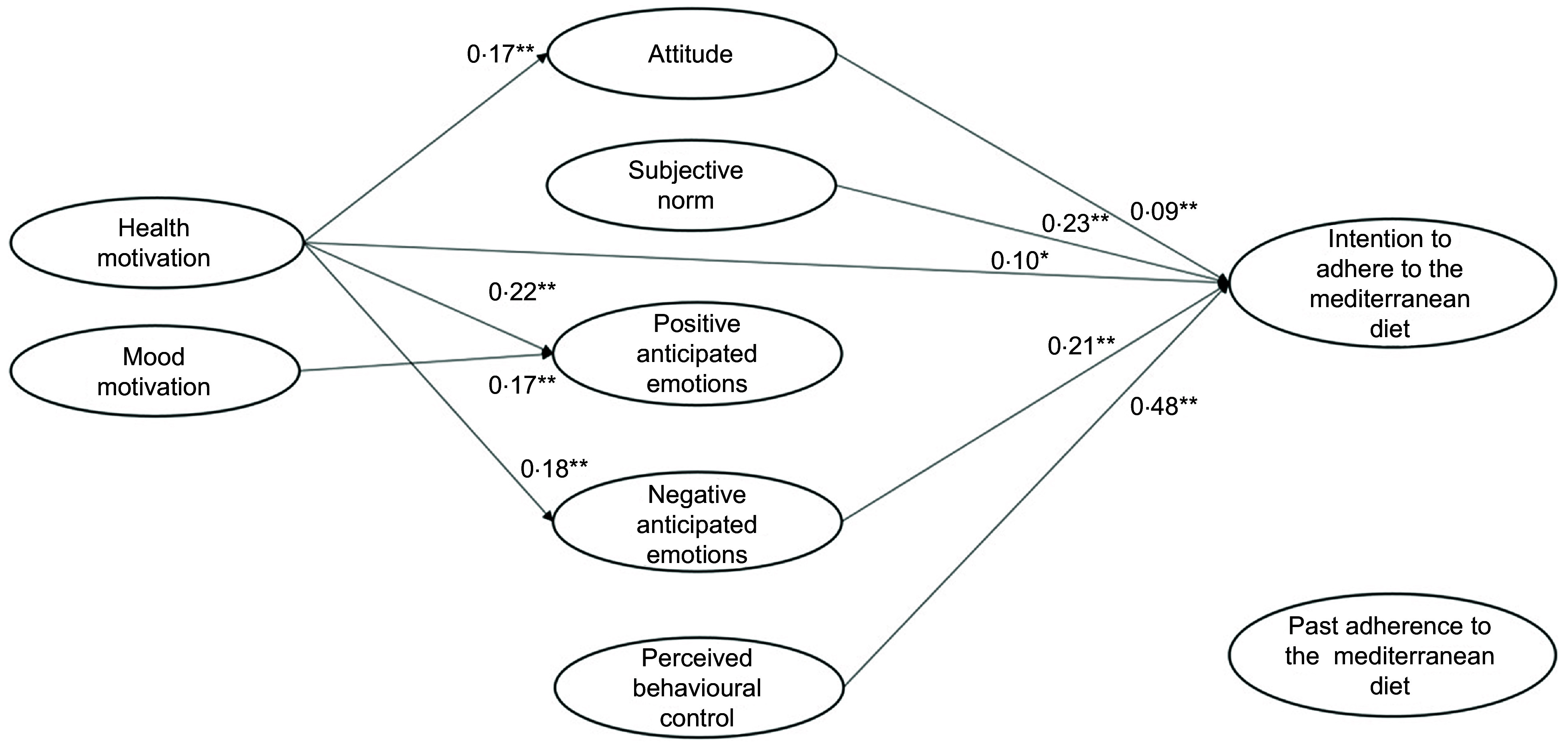
Low-adherence group: results of the integrated model to explain the intention to adhere to the Mediterranean diet

In the case of low past adherence to the MeDiet (Table 5, Fig. 3), participants’ perception of behavioural control and societal expectations about the adherence to the MedDiet were the most important antecedents of their intention to adhere to it, followed by negative anticipated emotions and attitude. Health motive had a direct effect on intention. Moreover, this motivational determinant indirectly increased participants’ intention by leveraging on their anticipation of negative emotions deriving from missed adherence to the MeDiet. Mood motive, instead, had only an effect on the anticipation of positive emotions deriving from adhering to the MeDiet. However, this motive did not impact on intention to adhere to the MeDiet. Finally, participants’ past adherence did not explain their intention.

**Fig. 4:**
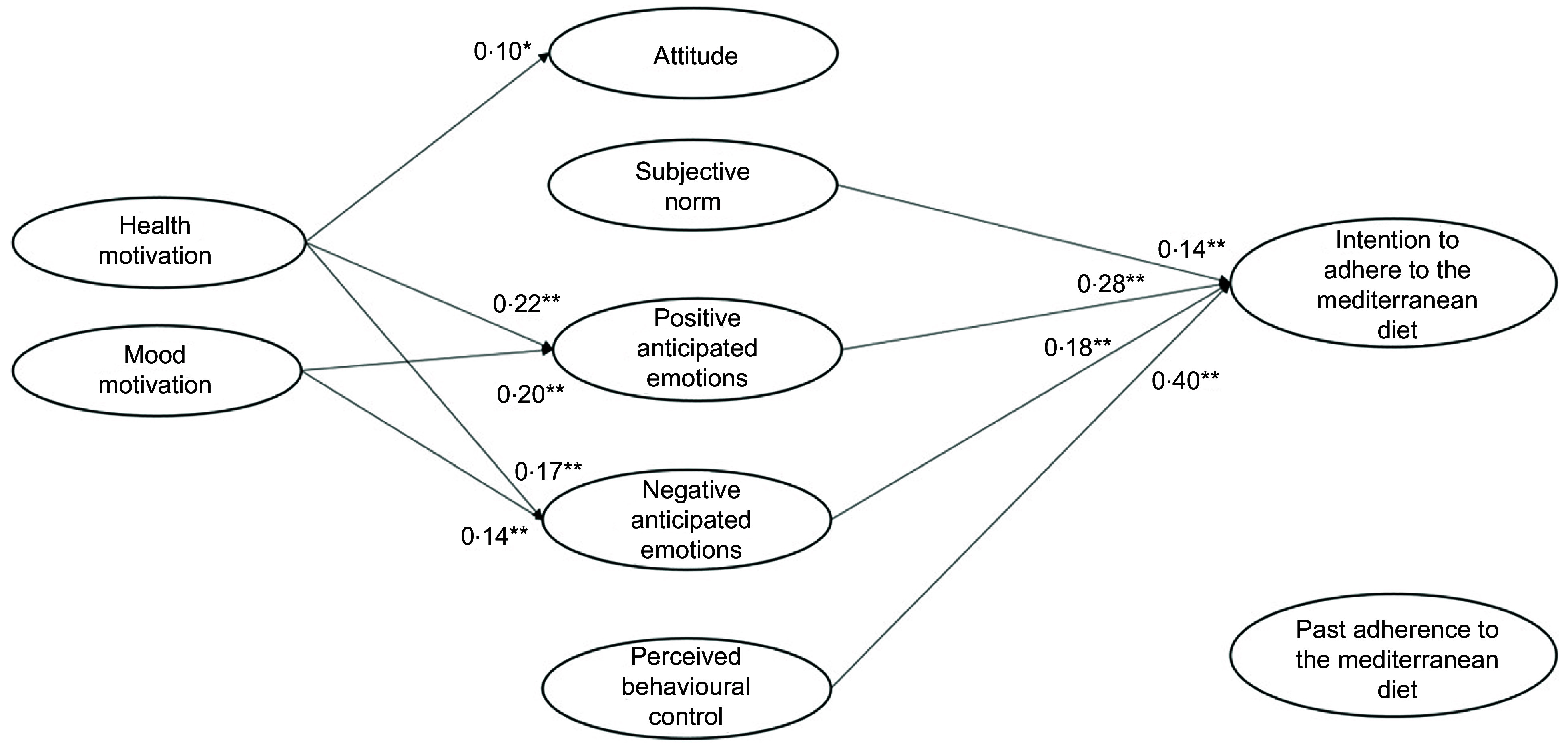
Medium-adherence group: results of the integrated model to explain intention to adhere to the Mediterranean diet

In the case of medium past adherence to the MeDiet (Table 5, Fig. 4), participants intended to follow it when they had a high perception of control and high positive and negative anticipated emotions. In this group, the role of subjective norm was low, and the role of attitude was NS. In addition, the health and mood motives exerted an impact on participants’ intention only if they elicited the anticipation of positive and negative emotions related to adherence or not adherence to the MeDiet. Finally, past adherence to the MeDiet increased participants’ intention to adhere to the MeDiet in the next month.

**Fig. 5:**
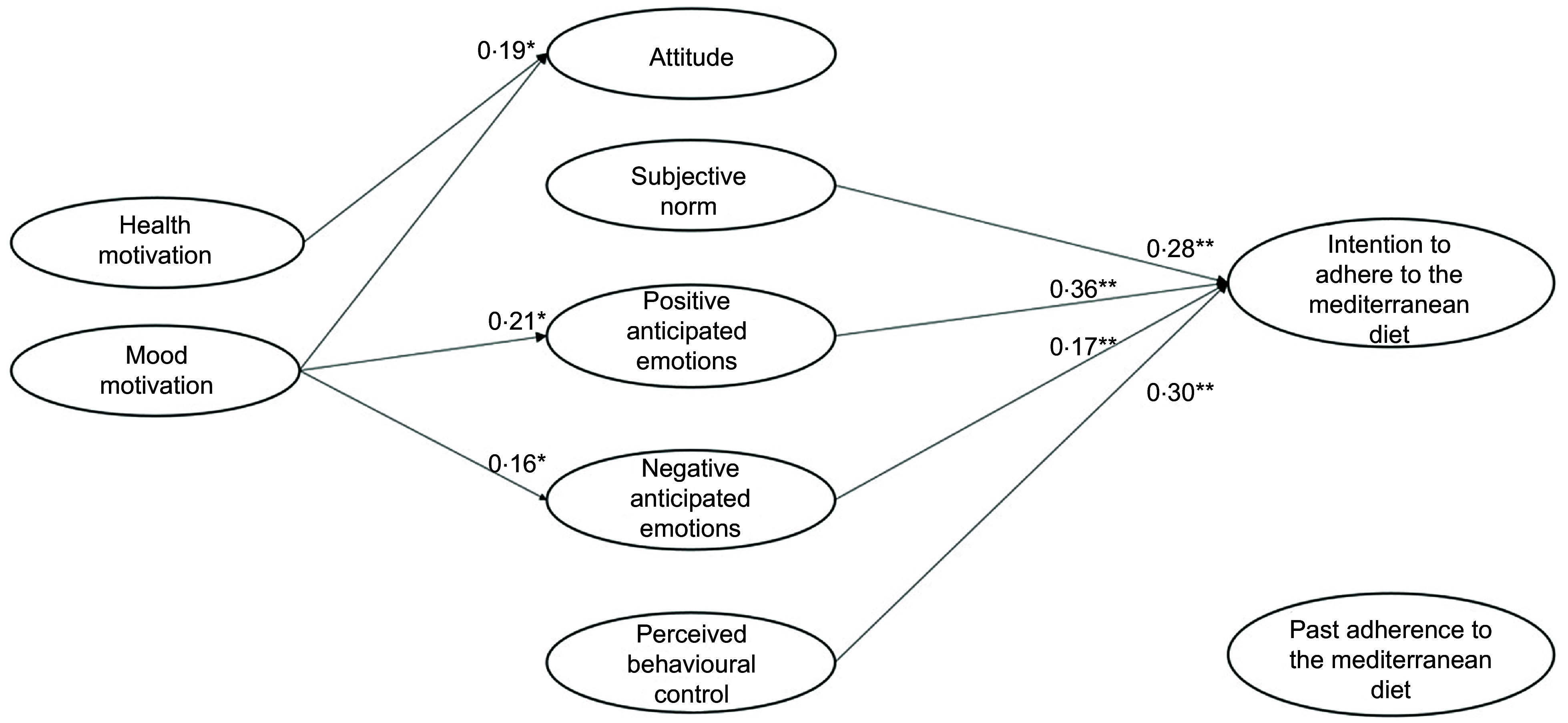
High-adherence group: results of the integrated model to explain intention to adhere to the Mediterranean diet

Finally, in the case of participants with a high past adherence to the MeDiet (Table 5, Fig. 5), emotional antecedents played a decisive role. These participants intended to keep following this diet mainly because they anticipated positive and negative emotions if they would engage (or not) in this behaviour in the future. They were also influenced by high perceptions of control over the behaviour and social expectations about it. Again, participants’ attitude towards adhering to the MeDiet did not influence their intention. Interestingly, among the motivational antecedents only the mood motive had an important influence on intention, given its ability to elicit the anticipation of positive/negative emotions. Health motive, instead, did not play a relevant role.

### Tests of invariant paths in the multigroup SEM model

Table 6 reports the findings of the Wald tests for each comparison used to disconfirm the invariance of the paths among study variables across groups. In these analyses, we run the Wald only when a path was significant in at least one group. The main results are discussed below.

**Table 6:** Results of the comparisons of the main paths among participants’ levels of adherence to the Mediterranean diet (MeDiet)

	Low adherence *v*. medium adherence	Low adherence *v*. high adherence	Medium adherence *v*. ‘rence
a. Attitude towards the MeDiet → intention to adhere to the MeDiet	*χ*^2^ (1) = 1·83, *P* = 0·17	*χ*^2^ (1) = 1·73, *P* = 0·19	*χ*^2^ (1) = 0·20, *P* = 0·67
b. Perceived behavioural control → intention to adhere to the MeDiet	*χ*^2^ (1) = 3·09, *P* = 0·05	*χ*^2^ (1) = 3·98, *P* = 0·05	*χ*^2^ (1) = 0·66, *P* = 0·42
c. Subjective norm → intention to adhere to the MeDiet	*χ*^2^ (1) = 4·06, *P* = 0·05	*χ*^2^ (1) = 0·02, *P* = 0·88	*χ*^2^ (1) = 2·69, *P* = 0·10
d. Positive anticipated emotions → intention to adhere to the MeDiet	*χ*^2^ (1) = 14·00, *P* = 0·001	*χ*^2^ (1) = 12·83, *P* = 0·001	*χ*^2^ (1) = 2·79, *P* = 0·05
e. Negative anticipated emotions → intention to adhere to the MeDiet	*χ*^2^ (1) = 0·49, *P* = 0·48	*χ*^2^(1) = 0·78, *P* = 0·38	*χ*^2^(1) = 0·11, *P* = 0·73
f. Health motive → attitude towards the MeDiet	*χ*^2^ (1) = 1·39, *P* = 0·24	*χ*^2^ (1) = 0·14, *P* = 0·71	*χ*^2^ (1) = 1·26, *P* = 0·26
g. Health motive → intention to adhere to the MeDiet	*χ*^2^ (1) = 1·85, *P* = 0·17	*χ*^2^ (1) = 1·92, *P* = 0·16	*χ*^2^ (1) = 0·35, *P* = 0·56
h. Health motive → positive anticipated emotions	*χ*^2^ (1) = 0·37, *P* = 0·54	*χ*^2^ (1) = 0·37, *P* = 0·54	*χ*^2^ (1) = 0·47, *P* = 0·49
i. Health motive → positive anticipated emotions → intention to adhere to the MeDiet	*χ*^2^ (1) = 8·08, *P* = 0·00	*χ*^2^ (1) = 1·13, *P* = 0·29	*χ*^2^ (1) = 0·06, *P* = 0·81
l. Health motive → negative anticipated emotions	*χ*^2^ (1) = 0·07, *P* = 0·79	*χ*^2^ (1) = 0·07, *P* = 0·80	*χ*^2^ (1) = 0·02, *P* = 0·89
m. Mood motive → negative anticipated emotions → intention to adhere to the MeDiet	*χ*^2^ (1) = 0·32, *P* = 0·57	*χ*^2^ (1) = 0·35, *P* = 0·55	*χ*^2^ (1) = 0·06, *P* = 0·81
n. Mood motive → positive anticipated emotions	*χ*^2^ (1) = 0·72, *P* = 0·40	*χ*^2^ (1) = 0·15, *P* = 0·70	*χ*^2^ (1) = 0·06, *P* = 0·80
o. Mood motive → positive anticipated emotions → intention to adhere to the MeDiet	*χ*^2^ (1) = 9·93, *P* = 0·00	*χ*^2^ (1) = 3·98, *P* = 0·05	*χ*^2^ (1) = 0·08, *P* = 0·77
p. Mood motive → negative anticipated emotions	*χ*^2^ (1) = 2·24, *P* = 0·13	*χ*^2^ (1) = 1·31, *P* = 0·25	*χ*^2^ (1) = 0·05, *P* = 0·81
q. Mood motive → negative anticipated emotions→ intention to adhere to the MeDiet	*χ*^2^ (1) = 1·18, *P* = 0·28	*χ*^2^ (1) = 0·67, *P* = 0·41	*χ*^2^ (1) = 0·00, *P* = 0·96

Considering the rational antecedents of the adherence to the MeDiet, the Wald test confirmed that a positive attitude towards the MeDiet had a direct effect on participants’ intention only in the case of low adherence to the MeDiet (Table 6, a). The impact of perceived behavioural control was instead higher when people had a low adherence to the MeDiet, as compared with people with a medium or high adherence (Table 6, b). As to social antecedents, Wald results confirmed that in the medium-adherence group, the societal expectation had a lower impact on intention than in the low-adherence group, while the other comparisons were not significant (Table 6, c).

As for the role of the emotional antecedents, positive anticipated emotions did not influence intention when participants had a low past adherence, while their effect increased as past adherence increased (Table 6, d). Differently, negative anticipated emotions had the same role in determining participants’ intention in all three groups (Table 6, e).

If we now consider the motivational antecedents, we find that participants’ health motive had a greater indirect effect on intention through anticipated positive emotions in the case of the medium-adherence group, compared with the high-adherence group (Table 6, i). Meanwhile, the effect of mood motive on positive anticipated emotions was lower in the low-adherence group, compared with both the medium- and high-adherence groups (Table 6, n). Consistently, also its indirect effect on intention through positive anticipated emotions was absent in the low-adherence group but present in the other two groups (Table 6, o). The other Wald tests did not reveal further differences in the effect of motives on the other study variables (Table 6, f, g, h, l, m, p, q).

Finally, we did not find difference in the behavioural antecedent, given that it was not a significant factor in all three models.

## Discussion

The current study contributes to our understanding of the psychosocial antecedents determining Italians’ intention to adhere to the MeDiet. Specifically, we validated an integrated psychosocial framework to analyse the role of rational, social, emotional, motivational and behavioural antecedents of the adherence to the MeDiet and to verify if baseline adherence to MeDiet moderates the relationships among these antecedents. Our results add to previous literature in many respects.

As to the *rational* and *social antecedents* of the intention to adhere to the MeDiet, similarly to what found by Mari *et al.*^(13)^, our model showed that perceived behavioural control was the most important factor of the intention to adhere to the MeDiet. This result suggests that people need to feel sure about their own capability and possibility to follow the MeDiet to behave accordingly. Differently from the results of past research on individual food choice, in our study a positive attitude towards MeDiet had only no influence on intention and a lower impact as compared with the impact of subjective norm^(9)^. This result highlights the role of social context in determining Italians’ willingness to follow the MeDiet. Likely, the higher effect of the social expectation in the case of the MeDiet can be attributed to the important cultural heritage connected with this diet in Italy.

Our results add to the current literature on the role of emotional antecedents in the adherence to a healthy diet. Only few past studies have considered the role of both positive and negative anticipated emotions in developing food choice intention^(23)^ and these studies were focused on specific food choices, such as eating filled chocolate^(23)^ or hamburgers^(42)^. Therefore, our results add to the current literature showing that both positive and negative anticipated emotions are important antecedents of the intention not only in the case of specific food choices but also in the case of overall healthy dietary choices. Moreover, our findings show that positive anticipated emotions have a greater impact than negative anticipated emotions on Italians’ intention to adhere to the MeDiet.

In our study, anticipated emotions had a higher impact than attitude on intention. In line with Ajzen and Fishbein^(45)^, we considered attitude as the rational evaluation of both the experiential and instrumental consequences of the MeDiet. The former refers to the benefits and costs associated with this behaviour (e.g. healthy or unhealthy, foolish or wise). The latter is related to emotion-laden judgments about the consequences of this behaviour (e.g. pleasant or unpleasant, enjoyable or unenjoyable). The difference between the experiential component of attitude and the anticipated emotions lies in at least two aspects^(46)^. First, anticipated emotions focus on the affects that are expected to follow an action or inaction, rather than those expected to occur while the action is being performed. Second, anticipated emotions are self-conscious emotions (e.g. pride, regret…), while experiential attitudes are focused on hedonic emotions (e.g. pleasure, enjoyment…). Therefore, the Italian adherence to the MeDiet seems to be driven more by self-conscious emotions that are expected to follow the adhesion to it, rather than by contextual experiential or instrumental consequences.

As to *motivational antecedents*, in our study health motive determined both emotional and rational antecedents. Specifically, our study offers the first evidence that health motive influences both positive and negative anticipated emotions and has an indirect effect on intention through them. These results underline that Italians with health motive intend to adhere to the MeDiet to experience future self-conscious positive emotions and avoid negative self-conscious emotions, rather than to obtain experiential or instrumental consequences from adherence. Mood motive also influenced both positive and negative anticipated emotions related to the MeDiet and had an indirect effect on intention to adhere to it. Again, people who prefer food that increases their positive mood apparently do not adhere to the MeDiet because they evaluate its experiential or instrumental benefits. They are more likely to adhere because they anticipate that they will feel proud, relaxed or satisfied and will avoid experiencing regret, worry or dissatisfaction.

Finally, as regards *behavioural antecedents*, our results show that the addition of past behaviour increased the explanation of intention significantly. In the model including behavioural antecedents, rational, social and emotional antecedents had the same effect as the previous Model 3. However, health motive was no longer a significant predictor of intention, suggesting that in the case of recurrent behaviours the recall of the health motivational antecedent is not needed anymore.

Besides having a direct effect on intention, past behaviour moderated the relationships among the other study variables determining intention. First of all, as to *rational antecedents* perceived behavioural control influenced the intention to adhere to the MeDiet in all three groups. This suggests that, independently from the level of adherence, people intend to follow the MeDiet if they perceive that this choice is possible and easy to perform. As to positive attitude, in our study it influenced intention only in the case of a low adherence to the MeDiet, that is when individuals were not engaged in the behaviour yet. As regards *social antecedents*, subjective norm determined the intention to adhere to the MeDiet in all three groups, suggesting that Italians intend to follow the MeDiet if they perceive that the important others will approve this choice. As to *emotional antecedents*, they also impacted on the intention to adhere to the MeDiet in all three groups, with an exception for positive anticipated emotions in the low-adherence group. This latter result could be explained considering that individuals with a low adherence to the MeDiet have probably little experience of positive emotions for having followed the MeDiet. For this reason, they may not expect to experience them in the future, and thus they do not anticipate these emotions as a consequence of the adherence to the MeDiet. In contrast, individuals with medium or high adherence to the MeDiet have previously experienced positive emotions associated to their behaviour and therefore anticipate them during the decision-making process.

If we now turn our attention to *motivational antecedents*, we notice that health motive influenced attitude towards the MeDiet in all three groups. However, only in the low-adherence group health motive had an indirect impact on intention via attitude and negative anticipated emotions. Therefore, when people have followed the Mediterranean diet very little in the past, they are pushed to do so in the future for rational reasons and to avoid negative emotions. On the contrary, in the high-adherence group health motive had no impact on intention. Unlike health motive, mood motive increased its relevance with increasing participants’ adherence. Specifically, it had an indirect impact on intention through positive and negative anticipated emotions in the medium-adherence group, and through positive anticipated emotions in the high-adherence group. The effects of the MeDiet on mental wellbeing are less known than those on physical health, and individuals who follow the MeDiet constantly may be more informed about the psychological effects of following the MeDiet, or have previously experienced them.

In sum, the results of the present study suggest that rational antecedents are the most relevant predictors of the intention to adhere to the MeDiet in the case of low adherence to it, followed by negative anticipated emotions and health motive. Instead, positive anticipated emotions and mood motive play a more central role in maintaining the intention to follow the MeDiet when people already do so.

### Limitations

Although this study offers several insights on what the main antecedents of the MeDiet are and how they are influenced by past adherence, it is not exempt from some limitations. First, although our sample was large, distributed in a sizeable age range, and enough balanced for gender, it was not representative of the general Italian population, because it was not fully balanced in terms of other sociodemographic variables (e.g. the Italian region of residence, the level of education, the average income). Thus, our data should be generalised with caution and future studies could usefully test the explanatory power of this model in other populations. They could also usefully evaluate whether the relationships between the antecedents of the intention to adopt the MeDiet are different in populations living far from the Mediterranean basin.

Second, in our integrated model, we did not control for the role of socio-demographic variables, as well as other variables about sensory aspects (e.g. taste), and medical conditions. This limitation depended on the size of our sample, which limited the number of paths that we could include and led us to focus only on the most relevant factors within a psychosocial theoretical framework. Moreover, in this study we collected data in a single time, limiting our analyses on future intentions and behaviour of the previous week. Future studies could consider adopting a longitudinal design with two times, which would include a behavioural measurement at time 2 and allow considering to what extent the intention to adopt the MeDiet is translated into actual behaviour. This could also help understanding whether some of the considered antecedents at time 1 moderate the relationship between intention at time 1 and behaviour at time 2. Furthermore, in this study we did not include a measurement of the moral antecedents of participant’s intention to adhere to the MeDiet. People’s adherence to the MeDiet might be guided by pro-environmental values and the awareness of the environmental consequences of their food choices^(47,48)^. In a more extensive model of antecedents of the MeDiet adherence future studies might therefore include a moral dimension.

Third, in our study we did not consider participants’ knowledge of the MeDiet. Although the study was conducted in a Mediterranean country and we clearly defined the MeDiet at the beginning of the study (Appendix 1), participants’ knowledge of the MeDiet could influence the observed relationships among variables. Future studies could consider if participants’ knowledge of the MeDiet covariates with both socio-demographic and psycho-social variables in determining the intention to adhere to the MeDiet.

Last, but not least, this study has the common limitation of quantitative research in psychology. Statistical analysis not always allows a meaningful theoretical interpretation, given that participants may have differently interpreted the same items^(49)^. Using a mixed methods approach, future studies on MeDiet could integrate quantitative measures with qualitative ones (i.e. interview, focus group) to better investigate how people, characterised by different sociodemographic and psychosocial characteristics, subjectively interpret the determinants of the MeDiet investigated in this study.

### Practical implications

As to the practical implications deriving from our findings, the present study offers at least three important insights for future public actions and campaigns aimed at promoting greater adherence to the MeDiet. First, we found that perceived behavioural control was the most important antecedent for Italian participants, regardless of their past adherence to the MeDiet. In light of this result, policy-makers should focus on how to increase Italians’ perceptions of control towards selecting Mediterranean food. This could be achieved by providing information on how to recognise and include it in a balanced diet. For example, future public campaigns might propose alternative dishes and recipes to reduce unhealthy foods and replace them with healthier choices. Moreover, Italian institutions might work on making the MeDiet more accessible by reducing the taxation on local products or recommending their introduction in school or work canteens. Second, we found that the social context and related expectations induce Italians to choose a MeDiet. This is linked to two of the pillars of the MeDiet, which are commensality (i.e. the act of eating with other people) and conviviality (i.e. the pleasure associated to shared meals)^(50)^. Policy makers could enhance the perceived benefits of eating together, also by trying to reduce the economic, time and social pressures that may inhibit this pleasurable activity.

Third, our results demonstrate that, according to the degree of past adherence to the MeDiet, different psychosocial factors drive the choice of further adhering to it. Interventions and communications should therefore be tailored accordingly, to maximise their effect on promoting the MeDiet. Specifically, our findings suggest that enhancing positive attitudes towards the MeDiet could be a promising ‘blanket approach’ only with people who have a low past adherence to it. In this case, communication should highlight the positive instrumental and experiential consequences that can derive from Mediterranean food, such as the pleasant taste of it or its healthy properties. In the case of people who already widely adhere to the MeDiet, communication should instead leverage self-conscious positive emotions (such as pride and satisfaction), thus increasing the possibility that this healthy eating practice will be maintained over time.

Finally, future studies might explore the possibility of applying this theoretical model to the development of communication strategies useful for promoting the adherence to the MeDiet using machine learning. Based on our findings, social psychologists and engineers might build together a model of a dialogue manager capable of fast profiling recipients and selecting the messages that are potentially most persuasive according to recipients’ profiles^(51,52)^.

## Conclusion

The present research contributes to our understanding of the psychosocial antecedents associated with the Italians’ intention to adhere to the MeDiet. At first glance, such intention seems to be mostly influenced by perceived behavioural control and positive anticipated emotions, with some further indirect effects of motivational antecedents. However, we have seen that a more complex picture appears when considering participants’ different degrees of past adherence to the MeDiet. Our results therefore confirm recent arguments according to which a ‘one-size fits all’ strategy might not be the most effective approach for encouraging healthy eating behaviour.

## Supplementary material

For supplementary material accompanying this paper visit https://doi.org/10.1017/S1368980022000878. click here to view supplementary material
