# Supplementary material for: The psychosocial antecedents of the adherence to the Mediterranean diet
Source: Public Health Nutr. 2022 Apr 13;25(10):2742–57. doi: 10.1017/S1368980022000878 (PMC9991846; doi:10.1017/S1368980022000878)
Supplement: Supplementary file 1 [file S1368980022000878sup001.docx]

**The Psychosocial Antecedents of the Adherence to the Mediterranean Diet**

**Appendix**

**Definition of the MeDiet in the Questionnaire**

At Time 1, during the completion of the questionnaire, all participants read the definition of the MeDiet reported below.

*“This figure* (Figure 1) *represents the* ***Mediterranean Diet****, a dietary pattern linked to the traditional food styles of countries bordering the Mediterranean Sea. The Mediterranean Diet is a “transnational intangible heritage” of the following countries: Italy, Cyprus, Croatia, Greece, Morocco, Spain, and Portugal.*

*This dietary pattern is based on the consumption of:*

- *numerous plant foods (fruit, vegetables, bread, other cereals, potatoes, legumes);*
- *olive oil as the main source of fat;*
- *milk products and dairy products (mainly cheese and yogurt);*
- *fish and poultry in moderate quantities;*
- *up to four eggs per week;*
- *an extremely reduced quantity of red meat;*
- *a moderate quantity of wine mainly during meals.*

**Figure 1. Representation of the MeDiet in the Questionnaire.^[[1]](#footnote-1)^**


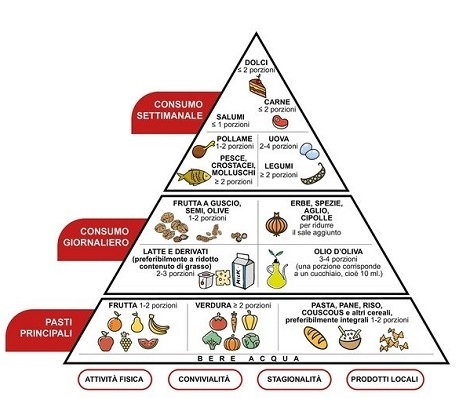


1. Retrieved from: <https://www.fondazioneveronesi.it/magazine/articoli/alimentazione/dieta-mediterranea-una-piramide-di-salute> [↑](#footnote-ref-1)
